# Assessment of complementary feeding of Canadian infants: effects on microbiome & oxidative stress, a randomized controlled trial

**DOI:** 10.1186/s12887-017-0805-0

**Published:** 2017-02-14

**Authors:** Wafaa Qasem, Meghan B. Azad, Zakir Hossain, Elnaz Azad, Sarah Jorgensen, Sandra Castillo San Juan, Chenxi Cai, Ehsan Khafipour, Trust Beta, L. Jackson Roberts, James Friel

**Affiliations:** 1 Departement of Human Nutritional Sciences, University of Manitoba, Winnipeg, MB Canada; 2 Richardson Centre for Functional Foods and Nutraceuticals, 196 Innovation drive, University of Manitoba, Winnipeg, MB Canada R3T 6C5; 3 Department of Pediatrics & Child Health, University of Manitoba, Winnipeg, MB Canada; 4 Children’s Hospital Research Institute of Manitoba, Winnipeg, MB Canada; 5 Department of Animal Science, University of Manitoba, Winnipeg, MB Canada; 6 Department of Medical Microbiology, University of Manitoba, Winnipeg, MB Canada; 7 Department of Food Science, University of Manitoba, Winnipeg, MB Canada; 8 Department of Pharmacology, Vanderbilt University School of Medicine, Nashville, TN USA

**Keywords:** Breastfed infants, Complementary feeding, Iron fortified cereal, Meat, Microbiome

## Abstract

**Background:**

The World Health Organization recommends exclusive breastfeeding until 6 months followed by introduction of iron-rich complementary foods (CFs). The aim of this study was to determine the impact of different iron-rich CFs on infant gut inflammation and microbiota.

**Methods:**

Eighty-seven exclusively breastfed infants were randomly assigned to receive one of the following as their first CF: iron-fortified cereal (Cer), iron-fortified cereal with fruit (Cer + Fr), or meat (M). Urine and stool samples were collected to assess reactive oxygen species (ROS) generation, gut microbiota and inflammation.

**Results:**

Fecal iron differed across feeding groups (*p* < 0.001); levels were highest in the Cer group and lowest in M group. A significant increase of fecal ROS formation (*p* < 0.002) after the introduction of CFs was observed, but did not differ across feeding groups. Fecal calprotectin increased within all groups after the introduction of CFs (*p* = 0.004). Gut microbiota richness increased after introduction of M or Cer + Fr. Regardless of feeding group, Coriobacteriaceae were positively correlated with ROS and Staphylococcaceae were negatively correlated with calprotectin.

**Conclusions:**

Choice of first CF may influence gut inflammation and microbiota, potentially due to variations in iron absorption from different foods. Further research is warranted to fully characterize these associations and to establish implications for infant health. This study was registered in the ClinicalTrial.gov registry (Identifier No. NCT01790542).

**Trial registration:**

This study was registered in the ClinicalTrial.gov registry under the name “Assessment of Complementary Feeding of Canadian Infants” (Identifier No. NCT01790542) February 6, 2013.

**Electronic supplementary material:**

The online version of this article (doi:10.1186/s12887-017-0805-0) contains supplementary material, which is available to authorized users.

## Background

By 6 months of age, breastfed infants become dependent on complementary foods (CFs) as a source of iron to prevent iron deficiency (ID), as a result of declining iron stores present at birth and low levels of iron in breast milk [1]. The World Health Organization and Health Canada recommend the introduction of meats and traditional iron-fortified cereal as first CFs in order to meet the iron requirements of growing infants and to prevent ID and iron deficiency anemia (IDA). Iron deficiency and IDA can have devastating effects on neurodevelopment [2, 3]. In North America, iron is provided most commonly through iron-fortified cereals [4]. These cereals contain non-heme electrolytic iron, which is absorbed at a rate of < 5% [5]. Meat provides more readily absorbed heme iron with an absorption rate of 35% [6], yet, we reported previously that meat is among the least common CFs introduced to Canadian infants [4].

Available evidence suggests an association between excessive iron exposure in the intestinal tract and initiation of the inflammatory process by reactive oxygen species (ROS) generation [7, 8]. With such a high amount of iron in fortified cereals and low absorption rates, concerns have been raised about the possibility of the unabsorbed excess iron in these CFs causing ROS generation and inflammation in the large intestine of infants [9].

Cumulative evidence indicates that anti-oxidant rich foods, such as fruits prevent ROS generation, inflammation and disease initiation [10]. A study by Orozco et al. conducted on adults who received a high iron dosage of 120 mg/d has implicated iron in oxidative stress and inflammation [11], which was ameliorated with a concurrent administration of an antioxidant supplement. Infants introduced to iron-fortified cereals are receiving an equivalent dose of iron per kilogram body weight to adults in the Orozco et al. study and may be producing ROS, leaving them prone to inflammation [12, 13].

The human gut harbors a complex microbial ecosystem consisting of more than 1000 microbial species that contribute to host metabolism, endocrine signaling and immunity [14]. Dysbiosis of the infant gut microbiota may have long-term health implications including increased risk of allergic disease, obesity and other inflammatory and gastrointestinal disorders [15]. Animal studies suggest that iron supplementation may alter composition of the gut microbiota [16], but only two human studies have addressed this potential association in infants [17, 18].

To date, no studies have considered the safety of iron-fortified cereal (traditional) and meat from the ROS generation and inflammatory perspective and few studies have assessed the effect of iron fortification on the gut microbiome of breastfed infants. Therefore, the aim of this study was 1) to assess and determine the safety of the traditional and the recently recommended first CFs in regards to ROS generation and inflammation, 2) to determine if the presence of antioxidant (fruit) in the iron fortified cereal reduces the oxidative effect of iron in the intestinal tract, 3) and to determine the impact of different recommended CFs on infant gut microbiota.

## Methods

**Fig. 1:**
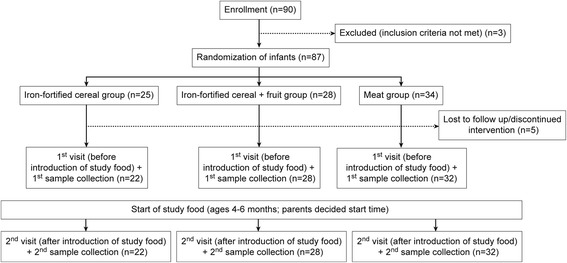
Study design

Eighty-seven full term healthy exclusively breastfed (EBF) infants were randomly assigned to one of three study foods: iron-fortified cereal (Cer), iron-fortified cereal with fruit (Cer + Fr), or meat (M). The random allocation sequence for the three feeding groups was generated using computer generated random numbers. This was done by the statistician of the Children’s Hospital Research Institute of Manitoba, Winnipeg, Manitoba using SAS/STAT software (SAS Institute Inc., Cary, NC, USA). Written informed consent was obtained from all caregivers before participation. The caregivers of the participating infants were blinded to the allocation in this trial, however, not to the CF. A total of 90 infants were assessed for eligibility. Three infants were excluded and the remaining 87 were enrolled in the study between December 2012 and May 2014 (25 in Cer group, 28 in Cer + Fr group and 34 in M group). The foods were the first CFs introduced to the infants and were consumed for a period of 2–4 weeks (Fig. 1). The study consisted of two visits in which urine, stool, dietary records and growth measurements were collected. In addition, a questionnaire was completed at the first visit to obtain socio-demographic characteristics and infant feeding patterns. Nutrient intakes were obtained using 3-day food records completed by caregivers. Nutrient intakes, socio-demographics, feeding patterns and infant growth were secondary outcomes that will be published in details elsewhere. The study was approved by the Bannatyne Campus Research Ethics Board, University of Manitoba and the Winnipeg Regional Health Authority Research Review Committee. The study was registered in the ClinicalTrial.gov registry (Identifier No. NCT01790542).

### Inclusion criteria

Infants who fullfilled the following inclusion criteria were considered eligible for participation: full term (>37 gestational weeks), birth weight of > 2500 g, absence of congenital anomalies and medical conditions, EBF at 4 – 6 months of age. Infants were excluded if they had started on complementary foods (other foods or liquids than breast milk) or consumed formula.

### Study foods

The three commercially available CFs (purchased) were as follows: iron-fortified rice cereal (Milupa GmbH, Danone, Friedrichsdorf, Germany), iron-fortified cereal with raspberry, pureed beef (Heinz, Pittsburgh, PA, USA). The nutritional compositions of the study foods are illustrated in Table 1. Iron-fortified rice cereal was selected because it was found to be the most common first CF choice among Canadian infants. Iron fortified rice cereal with fruit (raspberry) was selected because of the following: it is another commonly consumed infant cereal, contains a single fruit extract (raspberry powder) ingredient, raspberry is a rich source of phenolic phytochemicals and exhibits high antioxidant activities. Pureed beef infant food was selected for the following reasons: it contains single meat product (beef), red meat is a good source of heme iron, and it is recommended by Health Canada.

**Table 1:** Nutritional composition of study foods

	Iron fortified rice cereal Per 28 g (7–8 tbs)	Iron fortified rice cereal and fruit Per 28 g (7–8 tbs)	Meat Per 100 ml (1 jar)
Energy (Kcal)	120	120	140.9
Protein (g)	4.0	3.0	12.0
Carbohydrate (g)	21.0	21.0	0.3
Fibre (g)	0.0	0.0	0.3
Sugar (g)	3.0	4.0	0.0
Fat (g)	2.5	2.5	10.5
Cholesterol (mg)	ND	ND	29.4
Vitamin A (μg)	0.0	0.0	5.1
Vitamin B12 (μg)	0.35	0.17	1.4
Vitamin B6 (mg)	ND	ND	0.1
Thiamin (mg)	0.36	0.27	0.1
Riboflavin (mg)	0.38	0.38	0.2
Niacin (NE)	0.20	0.20	3.8
β Carotene (μg)	ND	ND	55.3
Vitamin C (mg)	0.00	0.00	0.0
Vitamin D (IU)	ND	ND	10.1
Vitamin E (IU)	ND	ND	ND
Sodium (mg)	20.0	20.0	38.5
Potassium (mg)	ND	ND	190.5
Calcium (mg)	30.3	20.0	4.1
Iron (mg)	8.80	8.80	1.3
Zinc (mg)	0.09	0.07	ND
Copper (μg)	13.2	13.2	ND
Magnesium (μg)	11.2	15.0	ND
Phosphorus (mg)	41.2	41.2	92.2
Iodine (μg)	13.0	13.0	ND
Manganese (mg)	0.04	0.00	ND
Selenium (μg)	ND	ND	2.7

Nutritional composition was calculated using the nutritional analysis software FoodFocus (Winnipeg, MB, Canada) and from food labels

*NE* niacin equivalent, *ND* no data provided, *tbs* table spoon

### Nutrient and breast milk intakes

The manufacturer presented the nutritional content of the food in % daily value of the recommended intakes (Dietary Reference Intakes, DRIs) rather than units, therefore, we converted the % values to the amounts in scientific units using the DRIs [19]. Dietary Iron and nutrient intakes data from the food records were calculated using the nutritional analysis software FoodFocus version 4 (Winnipeg, MB, Canada). Parents decided when to begin feeding and reported this date. Breast milk volume intake was estimated for each feeding duration recorded. For each minute, an estimated amount of 12.5 ml was given and up to 125 ml for full feeding lasting 10 min or longer [20, 21].

### Growth

The growth of the infants (weight, height and head circumference) was measured using standard techniques. Three measurements were taken for each variable and the values were averaged.

### Laboratory methods

#### Sample collection

Urine samples were collected by the infants’ caregivers using the pediatric urine collector (Kendall pediatric urine collector, Covidien, Mansfield, MA, USA) provided by the study team along with instructions. Fecal samples were collected from soiled diapers provided by the caregivers and transferred to plastic collection tubes by the study team. Urine and fecal samples were transported by the study team using a cooler with ice packs, separated in labeled 1.5 ml tubes and stored at -80 °C until analysis.

#### Urinary F_2_-Isoprostanes

F_2_-Isoprostanes are prostaglandin-like compounds produced as a result of free radical lipid peroxidation of arachidonic acid [22]. Levels of urinary F_2_-isoprostanes were determined by the liquid chromatography-mass spectrometry (LC/MS/MS) assay method developed by Davies et al [23]. Urinary creatinine concentrations were determined using the Creatinine Parameter Assay kit (R&D Systems Inc., Minneapolis, MN, USA) and following the manufacture’s guidelines. We expressed F_2_-isoprostane levels relative to urinary creatinine levels to normalize for excretion rate.

#### Fecal iron

Stool samples (300–400 mg) were digested by acid mix (6 M 37% HCL, 20% v/v trichloroacetic acid, Fisher Scientific Inc., MA, USA) to extract the fecal iron [11]. After incubation at 65 °C for 20 h, the fecal iron content was measured using the commercially available spectrophotometrically-based assay Feren-B kit (Bioanalytic, Umrich, Freiburg, Germany).

#### Fecal reactive oxygen species

High performance liquid chromatography (HPLC) was used to quantify and separate the end products of hydroxyl radical attack on salicylic acid, and resultant 3 products, particularly, 2,5 dihydroxybenzoic acid, 2,3 dihydroxy benzoic acid, and catechol using a method validated by Orozco et al [11]. and adapted from Owen et al [24]. Initially, a calibration curve was constructed using the standards of salicylic acid, 2,5 dihydroxybenzoic acid, 2,3 dihydroxybenzoic acid, and catechol. Fecal samples (100 mg) were incubated (100 mM phosphate buffer, 500 mM ethylenediaminetetraacetic acid (EDTA), 50 μM FeCL_3_H_2_O, and 2 mM salicylic acid) (Sigma-Aldrich, St. Louis, MO, USA) at 37 °C for 21 h. Following incubation, each sample was filter-sterilized and 20 μl of each sample was injected directly onto the HPLC column (ODS Hypersil 200 x 2.1 Thermo Fisher Scientific Inc.). For the chromatographic separation of compounds, the mobile phase comprised of 2% acetic acid glacial in water (solvent A) and methanol making up solvent B utilizing the following gradient: 95% A/5% B for 2 min, 75% A/25% B for 8 min, 60% A/40% B for 10 min, 50% A/50% B for 10 min and 0% A/100% B for 10 min. For the first 5.5 min, the UV/VIS detector was adjusted at 278 nm and changed to 301 nm until completion of the run. The optimal flow rate was 0.5 ml/min. Data handling and instrument control were conducted by the software ChemStation (Agilent ChemStation, Agilent Technologies, Santa Clara, CA, USA).

#### Fecal calprotectin

Fecal calprotectin concentrations were determined using the commercially available Calprotectin ELISA kit (BUHLMANN Calprotectin ELISA, ALPCO, Salem, NH, USA) and following the manufacture’s guidelines.

#### Fecal microbiota

Bacterial DNA was extracted from stool samples using the ZR fecal DNA kit (Zymo Research, Irvine, CA, USA), which included a bead-beating step for the mechanical lysis of the microbial cells. DNA was quantified using a NanoDrop 2000 spectrophotometer (Thermo Scientific). DNA samples were normalized to 20 ng/μl, and quality checked by PCR amplification of the 16S rRNA gene using universal primers 27 F (5’-GAAGAGTTTGATCATGGCTCAG-3’) and 342R (5’-CTGCTGCCTCCCGTAG-3’) as described previously [25]. Amplicons were verified by agarose gel electrophoresis. The V3-V4 region of the 16S rRNA gene was amplified using modified F515/R806 primers [26] as described before [27]. The reverse PCR primer was indexed with 12-base Golay barcodes, allowing for multiplexing of samples. For each sample, the PCR reaction was performed and the V3-V4 library was generated and quantified as described [25]. The 300 paired-end sequencing reaction was performed on a MiSeq Ilumina platform at the Gut Microbiome and Large Animal Biosecurity Laboratories. Paired-end sequences were merged using the PANDAseq assembler and standard QIIME pipelines were used to filter singletons [27, 28], remove chimeric reads and assign sequences to operational taxonomic units (OTUs) through alignment with the Greengenes reference database (version 13_5) [29]. After cleaning and processing, a total of 3.3 million reads were retained (median 30,500 per sample, range 14,500–45,900), representing 5,202 unique OTUs. For subsequent analyses, data were rarefied to 14,500 sequences per sample.

### Statistical analysis

Statistical analyses of iron, ROS and inflammatory biomarker data were performed using the statistical software package IBM SPSS version 22.0 (IBM Corp. Armonk, NY, USA). To compare feeding group means at two different time points, repeated measures analysis of variance (ANOVA) was used. For pair-wise comparisons, the post hoc Bonferroni test was used. *P*-values <0.05 were considered statistically significant. Microbiome analyses were performed using QIIME version 1.9.0 (qiime.org) and SAS version 9.2 (SAS Institute Inc., Cary, NC, USA). The Chao1 estimator of species richness and the Shannon Diversity Index were calculated to evaluate microbiota diversity within samples; these indices were normally distributed and comparisons were made by ANOVA or two-sided *t*-test. Permutational analysis of variance (PERMANOVA) and principal coordinates analysis (PCoA) of unweighted UniFrac distance matrices were used to assess between-sample differences in microbiota diversity and community structures [30]. The OTUs relative abundances were compared between feeding groups by non-parametric Kruskal-Wallis or Wilcoxon rank sum test, with false discovery rate correction for multiple comparisons. Spearman correlations were used to explore associations of microbiota parameters with fecal iron, calprotectin and ROS. Effects were considered significant at *p* < 0.05. Trends were discussed at *p* < 0.1.

## Results

A total of 82 infants completed the study; 5 withdrew (did not receive allocated intervention) due to the following reasons: mothers refused to comply with the assigned study food (*n* = 3), moved to a different province (*n* = 1), or failed to respond to study communications (*n* = 1). The baseline characteristics of study infants are shown in Table 2. There were no statistical significant differences between the groups for these characteristics except for higher maternal age and the use of vitamin supplementation during breastfeeding in mothers of the M group.

**Table 2:** Baseline comparisons of the three study group’s characteristics

Variable	Cer	Cer + Fr	M	All groups
	(*n* = 22)	(*n* = 28)	(*n* = 32)	(*n* = 82)
Males (%)	44.0	48.3	54.8	49.4
Birth weight (g) mean ± SD	3437 ± 475	3693 ± 494	3544 ± 519	3563 ± 503
Length at birth (cm) mean ± SD	52 ± 2.8	52.5 ± 2.6	51.7 ± 2.7	52 ± 2.7
HC at birth (cm) mean ± SD	34.1 ± 2.0	34 ± 1.9	35.5 ± 1.8	34.7 ± 1.9
Gestation (wk) mean ± SD	39.8 ± 1.3	39.8 ± 1.7	39.4 ± 1.0	39.7 ± 1.4
Parity (%)				
1	32	27.6	35.5	31.8
2	36	41.4	51.6	43.5
≥ 3	32	30.8	12.9	24.8
Parity mean ± SD	2.0 ± 0.8	2.3 ± 1.5	1.8 ± 0.9	2.0 ± 1.1
Mean Maternal age (years)	31.1 ± 4.4	31.9 ± 4.1	34.5 ± 3.7*	32.6 ± 4.3
Mode of delivery (%)				
Vaginal	83.3	92.3	83.3	86.3
Caesarian	12.5	3.8	16.7	11.3
Other	4.2	3.8	0.0	2.5
Antenatal-postnatal complication (%)				
Yes	24	20.7	29	24.7
No	76	79.3	71	75.3
Fe supplementation during pregnancy (%)	20	31	25.8	25.9
Vitamin supplementation during pregnancy (%)	88	89.7	96.8	91.8
Vitamin supplementation during BF (%)	48*	62.1*	79.3*	63.9
Marital status (%)				
Married	88	93.1	93.5	91.8
Single	4.0	6.9	0.0	3.5
Divorced	0.0	0.0	0.0	0.0
Common-law	8.0	0.0	6.5	4.7
Maternal pre-pregnancy weight (Kg) mean ± SD	67 ± 14.7	66.6 ± 12.1	69.8 ± 14.7	67.9 ± 13.8
Maternal height mean ± SD	165 ± 6.3	166.3 ± 6.9	166.5 ± 7.5	166 ± 6.9
Maternal pre-pregnancy BMI mean ± SD	24.6 ± 4.4	24.1 ± 3.9	25.3 ± 5.3	24.7 ± 4.6
Duration of previous BF (months) mean ± SD	14.9 ± 8.9	12.3 ± 7.5	13.4 ± 6.2	13.5 ± 7.4
Maternal education (%)				
Primary & secondary	16	10.7	9.7	11.9
Post secondary	84	89.3	90.3	88.1
Pre-delivery working mothers (%)	76	85.7	96.8	86.9
Paternal weight (Kg) mean ± SD	88.4 ± 14.9	92 ± 18.8	87.9 ± 11.7	89.5 ± 15.2
Paternal height mean ± SD	179.5 ± 8.0	180 ± 7.1	180 ± 7.9	180 ± 7.6
Paternal BMI mean ± SD	25.1 ± 8.7	28.3 ± 5.3	27.0 ± 4.2	26.9 ± 6.1
Paternal education (%)				
Primary & secondary	16.0	3.8	3.2	7.3
Post secondary	84.0	96.2	96.8	92.7
No. of household occupants mean ± SD	3.9 ± 0.8	4.3 ± 1.5	3.9 ± 1.0	4.0 ± 1.2
No. of household children mean ± SD	1.8 ± 0.7	2.4 ± 1.5	1.9 ± 0.9	2.0 ± 1.1
Maternal smoking (%)	4.0	0.0	3.2	2.4
Infant Vitamin D supplementation (%)	96.0	100.0	90.3	95.3

*BF* Breastfeeding, *BM* breast milk, *: *p* < 0.05

The primary outcome was fecal ROS generation. According to Orozco et al., the mean of ROS was found to be 10% less in adults who received antioxidant supplements [11]. Therefore, minimum sample size required for each group was 25 (*N* = 75) which was set at a significance level (α) 0.05 with power (β = 5%) of 95%. Due to insufficient sample volumes, the number of infant samples available to be analyzed for each outcome varied. The sample numbers analyzed were as follows: 25 urine samples for F_2_-Isoprastanes analysis, 77 stool samples for fecal iron analysis, 66 stool samples for fecal ROS analysis, 43 stool samples for fecal calprotectin, and 56 stool samples for microbiome. Table 3 summarizes the biomarker measures of infants in the study groups.

**Table 3:** Summary of biomarker measures of infants in the three study groups

Biomarker (mean ± SE)	Cer		Cer + Fr		M		*P* (within groups)	*P* (between groups)
	Before CFs	After CFs	Before CFs	After CFs	Before CFs	After CFs		
Urinary F2-Isoprostane	*N* = 7	*N* = 7	*N* = 8	*N* = 8	*N* = 10	*N* = 10	0.3	0.5 (Cer vs M)
(ng/mg) (*n* = 25)	0.45 ± 0.81	0.52 ± 0.78	0.35 ± 0.47	0.41 ± 0.08	0.48 ± 0.14	0.68 ± 0.12		0.5 (Cer vs Cer + Fr)
Fecal iron	*N* = 22	*N* = 22	*N* = 26	*N* = 26	*N* = 29	*N* = 29	<0.001*	<0.001* (Cer vs M)
(Fe g/feces) (*n* = 77)	3.9 ± 0.37	5.6 ± 0.38	3.9 ± 0.32	4.7 ± 0.34	2.9 ± 0.25	3.7 ± 0.25		0.014* (Cer + Fr vs M)
Fecal ROS	*N* = 16	*N* = 16	*N* = 21	*N* = 21	*N* = 29	*N* = 29	0.002*	0.6 (Cer vs M)
(mmol/l) (*n* = 66)	0.024 ± 0.007	0.037 ± 0.006	0.014 ± 0.003	0.031 ± 0.005	0.023 ± 0.004	0.028 ± 0.003		0.3 (Cer vs Cer + Fr)
Fecal calprotectin	*N* = 14	*N* = 14	*N* = 19	*N* = 19	*N* = 10	*N* = 10	0.004*	0.07 (Cer vs Cer + Fr)
(mg/g feces) (*n* = 43)	111.0 ± 12	122.3 ± 13	93.73 ± 12	154.5 ± 23	108.1 ± 11	131.9 ± 18		0.9 (Cer vs M)

*: *P* < 0.05 by repeated measures ANOVA

### Dietary iron intake

The dietary iron intakes of the infants (Cer *n* = 22, Cer + Fr *n* = 28, M *n* = 32) are reported in Table 4. There was an increase in iron intake after the introduction of the CFs in all groups (*p* < 0.05).

**Table 4:** Dietary iron intake before and after introduction of complementary foods (CFs) in the three study groups

		Before CFs			After CFs	
Feeding group	Cer	Cer + Fr	M	Cer	Cer + Fr	M
	(*n* = 22)	(*n* = 28)	(*n* = 32)	(*n* = 22)	(*n* = 28)	(*n* = 32)
Iron (mg) (mean ± SE)	0.28 ± 0.01	0.28 ± 0.01	0.29 ± 0.01	16.3 ± 2.5^*^	21.5 ± 3.4^*^	0.86 ± 0.08^*,**^

Dietary Iron intake data from the 3-day food records was calculated using the nutritional analysis software FoodFocus© (Winnipeg, MB, Canada)

^*^ values superscript letters are significantly different intake within groups (over time) (*p* < 0.05) by repeated measures ANOVA

^**^ values superscript letters are significantly different between the groups (*p* < 0.05) by repeated measures ANOVA

### Breast milk and nutrient intake

After the introduction of CF, the amount of breast milk intake decreased significantly within groups (over time). However, there was no significant difference between the groups in the intake of breast milk. There was a significant difference in energy intake within groups (over time) except for the M group (Additional file 1: Table S1, Additional file 2: Table S2 and Additional file 3: Table S3). Carbohydrate intake was significantly lower in the M group (*p* = 0.04). There were no significant differences in most of vitamins and minerals intake within the groups (over time) or between the groups.

### Growth

There were no significant differences in growth rates between male infants and female infants. Over all, no significant differences were seen in growth rates between the feeding groups (data not shown).

### Urinary F_2_-isoprostanes

**Fig. 2:**
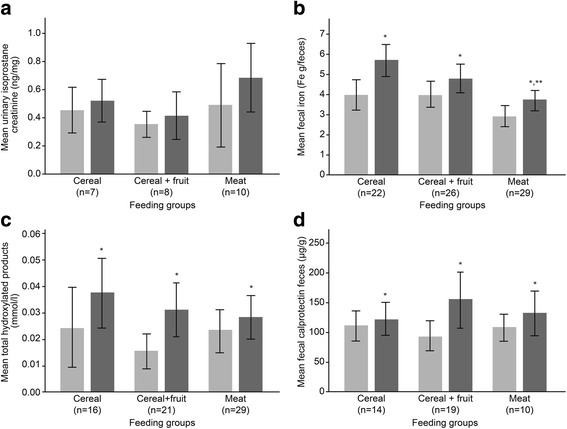
Comparisons between the feeding groups and over time for fecal iron, ROS, and inflammatory biomarkers at before and after introduction of complementary foods. Error bars indicate +/- 2 SE; comparisons by repeated measures ANOVA; (**a**) Average creatinine corrected F_2_-isoprostanes in urine (*n* = 25). No significant differences between groups or over time. **b** Fecal iron in the three study groups (*n* = 77).*Significant difference (*p* < 0.05) over time. **Significant difference between the groups. **c** Total hydroxylated products in the three study groups (*n* = 66). *Significant difference (*p* < 0.05) over time. **d** Fecal calprotectin concentration in the three study groups (*n* = 43). *Significant difference (*p* < 0.05) over time

The effect of the three foods on the excretion of the urinary F_2_-Isoprostanes (Cer *n* = 7, Cer + Fr *n* = 8, M *n* = 10) was not significant, although our sample size was small for this analysis (Fig. 2a).

### Fecal iron

Seventy-seven fecal samples (Cer *n* = 22, Cer + Fr *n* = 26, M *n* = 29) were analyzed for fecal iron (Fig. 2b). After the introduction of the CFs, there was an increase in the amount of fecal iron within groups (over time) (*p* < 0.001). There was also a feeding group effect (*p* < 0.001) with lower fecal iron levels in the M group (*p* < 0.001 vs. Cer and *p* = 0.014 vs. Cer + Fr).

### Fecal reactive oxygen species generation

In total, 66 fecal samples (Cer *n* = 16, Cer + Fr *n* = 21, M *n* = 29) were analyzed for formation of hydroxylated products, indicating the production of ROS (Fig. 2c). After the introduction of the CFs, there was an increase in ROS generation within groups (over time) (*p* < 0.05). No differences were found between groups.

### Fecal calprotectin

A total of 43 fecal samples (Cer *n* = 14, Cer + Fr *n* = 19, M *n* = 10) were analyzed to determine calprotectin concentration. There was a significant effect of time (*p* = 0.004) for fecal calprotectin level in all feeding groups (Fig. 2d). There was no significant difference observed between the CF groups. However, in post hoc analysis of the groups, fecal calprotectin of the Cer + Fr tended to be higher when compared to the Cer group (*p* 0.07).

### Fecal microbiota

**Fig. 3:**
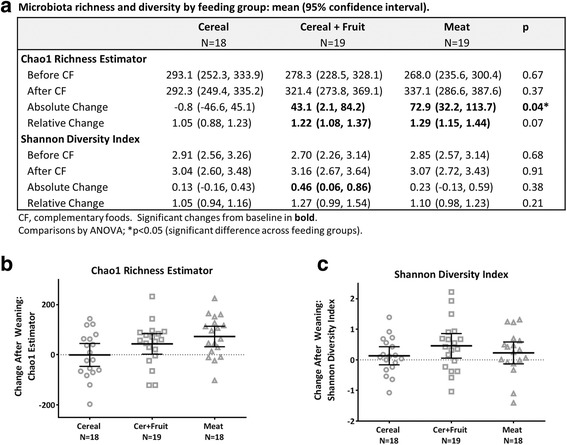
Change in microbiota richness and diversity after introduction of complementary foods. **a** Microbiota richness and diversity by feeding group. **b** Chao1 richness estimator. **c** Shannon diversity index. Bars indicate means with 95% confidence intervals. Between-group comparison by ANOVA. *Significant change from baseline (*p* < 0.05)

**Fig. 4:**
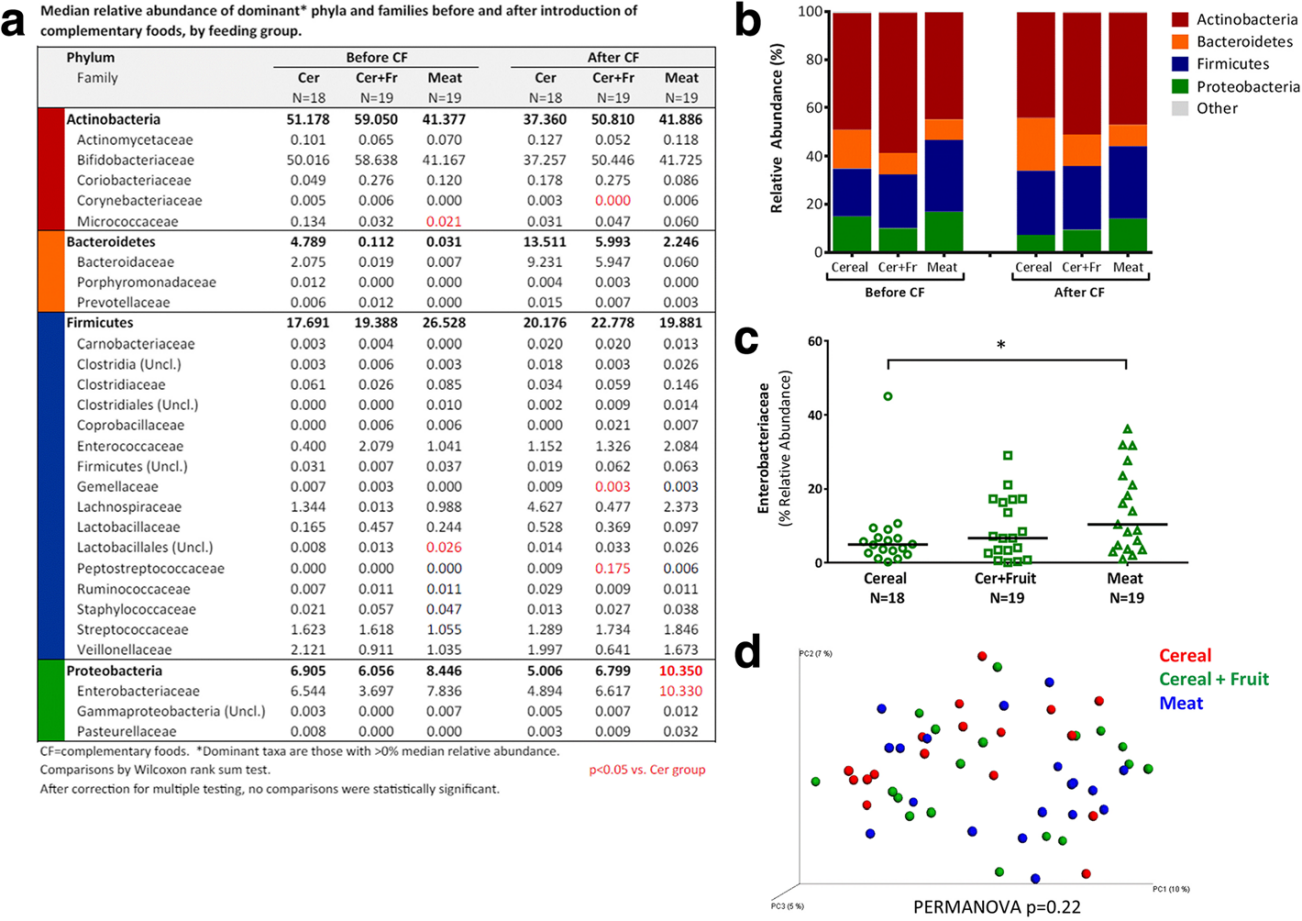
Gut microbiota composition before and after introduction of complementary foods (CFs). **a** Median relative abundance of dominant phyla and families before and after introduction of CFs, by feeding group. Dominant taxa are those with >0% median relative abundance. *Significant difference (*p* < 0.05) by Wilcoxon ranked sum test, compared to Cer group. **b** Mean relative abundance of dominant microbiota phyla by feeding group, before and after introduction of CFs. **c** Relative abundance of Enterobacteriaceae after introduction of CFs. Bars indicate medians. *Significant difference (*p* < 0.05) by Wilcoxon rank-sum test. **d** Principal components analysis of microbiota community structures (unweighted UniFrac distance) after introduction of CFs; statistical comparison by PERMANOVA with 500 permutations

**Fig. 5:**
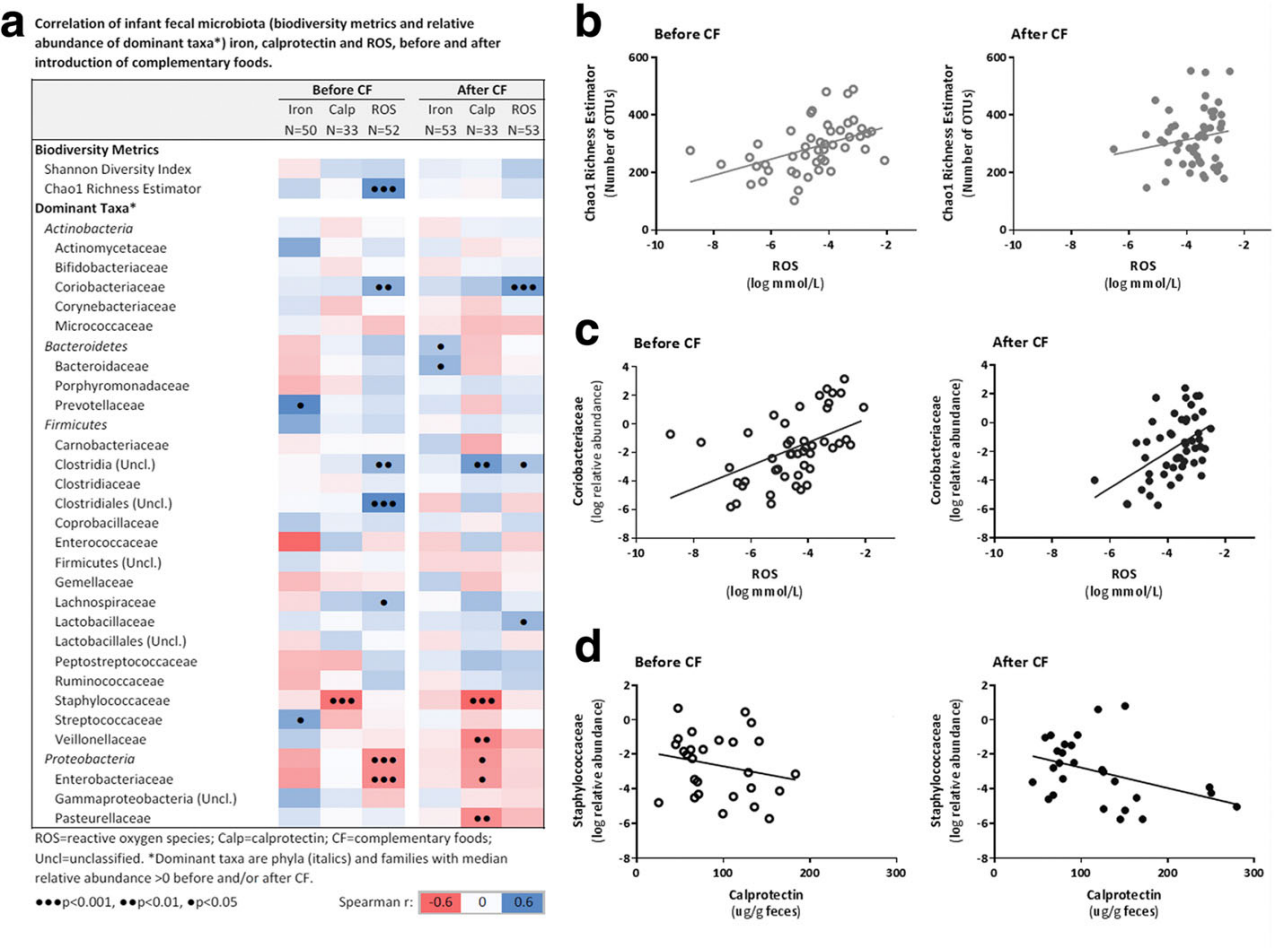
Correlations of fecal microbiota, iron, ROS and calprotectin, before and after introduction of complementary foods (CFs). **a** Spearman correlation matrix of dominant taxa (phyla and families with median relative abundance >0%) with fecal iron, ROS and calprotectin. **b** Correlation of microbiota richness and ROS, before CF: *r* = 0.51, *p* = 0.0001; after CF: *r* = 0.15, *p* = 0.29. **c** Correlation of Coriobacteriaceae and ROS, before CF: *r* = 0.39, *p* = 0.005; after CF: *r* = 0.46, *p* = 0.001. **d** Correlation of Staphylococcaceae and calprotectin, before CF: *r* = -0.54, *p* = 0.001; after CF: *r* = -0.59, *p* = 0.0003

Fecal microbiota was characterized in 56 infants (Cer, *n* = 18; Cer + Fr, *n* = 19; M, *n* = 19) before and after introduction of CFs. Gut microbiota richness increased following introduction of Cer + Fr or M, whereas no change was observed in the Cer group (Fig. 3). Across feeding groups, the relative abundances of dominant bacterial phyla and families were similar (Fig. 4a and b) and there were no clear differences in microbiota community structures (Fig. 4d, PERMANOVA *p* = 0.22). The median relative abundance of Bifidobacteriacea declined from 51% to 37% after introduction of iron-fortified Cereal, but remained relatively unchanged after introduction of meat (from 41% to 42%). Bacteroidetes increased with the introduction of CF across all feeding groups, reaching the highest levels (14%) in the Cer group. Enterobacteriaceae were overrepresented in the M group (Fig. 4c; median relative abundance 10.35% vs. 5.0% in the Cer group; *p* < 0.05); however, none of these differences were significant after correction for multiple comparisons. Some fecal microbiota parameters correlated with iron, ROS and calprotectin measurements (Fig. 5). Before introduction of CFs, microbiota richness was positively correlated with ROS (Spearman *r* = 0.51, *p* < 0.001), but there was no correlation after introduction of CFs (*r* = 0.15, *p* = 0.29) (Fig. 5a). Both before and after introduction of CFs, the relative abundance of Coriobacteriaceae was positively correlated with ROS (Fig. 5c), and the relative abundance of Staphylococcaceae was negatively correlated with calprotectin (all *p* < 0.005) (Fig. 5d).

## Discussion

This is the first RCT to investigate the effect of different first CFs on oxidative stress, inflammation and gut microbiota in healthy EBF infants. Several important findings emerge from the current study. Residual fecal iron was lower in the M group compared to the cereal groups. The urinary oxidative stress marker, F_2_-Isoprostane, did not change with introduction of CFs and was similar across the CF groups; however, the fecal oxidative stress marker of ROS production increased significantly within groups over time. Fecal calprotectin concentration increased significantly after the introduction of the CFs within groups. Finally, some gut microbiota differences were identified across feeding groups, and a number of associations with ROS and calprotectin were observed.

### Oxidative stress and inflammation

To our knowledge, no previous studies had evaluated the effect of different iron-fortified CF regimens on the oxidative stress status of EBF infants. A previous study conducted on three healthy adults who received daily 120 mg of oral iron for 7 days, showed that two of the subjects had a two-fold increase in urinary F_2_-Isoprostanes from the baseline [8]. Among the few studies that report normal F_2_-Isoprostanes levels in healthy children [22], there was an inverse association between age and the concentration of urinary F_2_-Isoprostanes (β coefficient: -0.14, *p* = 0.0001) [22]. In the current study the levels of urinary F_2_-Isoprostanes increased after the addition of the CFs, but these values remained in the normal range of urinary F_2_-Isoprostanes [22].

Our results are in keeping with those reported in a previous study conducted by Orozco et al. on seventeen healthy men who received daily 120 mg of iron for 2 cycles of 7 days supplementation. From that study, it was reported that after iron supplementation, there was a significant increase in fecal ROS production by 36% (*p* = 0.026) [11]. Similarly, in the current study, after the provision of iron-rich foods, ROS generation increased significantly by 55% (*p* = 0.003). Differences between these studies may be explained by the differences between adult and infant intestinal iron absorption. Previous studies have suggested that the dietary regulator of iron absorption between 6 and 9 months of age is immature and remains under development [31]. An additional difference between the adult study by Orozco et al and the current study is the form of the iron administered, which was a single supplement for the adults vs a poorly absorbed elemental iron fortificant in the infant CF. Another possible cause is the individual variation in the iron stores, which might have influenced the absorption rate thus affecting the amount of intestinal residual iron and ROS production [32]. In the current study, differences between groups in ROS generation were not significant despite differences in energy intake between the two cereal groups versus the M group. This finding suggest that ROS generation in response to feeding is an expected process in the under developed intestinal tract of the growing infants regardless of the type and amount of food. Another important factor that is noteworthy to mention is the phytate content of the iron-fortified cereals that might have interfered with iron absorption [33].

Our findings did not support our hypothesis that fruit added to iron-fortified cereal would ameliorate ROS generation in the intestinal tract. Rather, our results suggest that the average daily iron intake from Cer + Fr of 21.5 ± 3.4 mg (67.5 g of cereal/d) was enough to increase the production of ROS regardless of the fruit content. The low content of raspberry powder (1.8%) in the Cer + Fr was insufficient to eliminate the oxidative effects. In addition, it is possible that the raspberry powder may have lower antioxidant activity compared to fresh raspberry fruit, due to variations in drying techniques and processing.

In the current study differences in the baseline of both residual fecal iron and ROS generation were observed before the introduction of solids. Although all of the included infants were EBF, the variation in the use of multivitamin supplements by the mothers, genetic factors, the possibility of formula consumption, and analytical errors might have contributed to these observations [34, 35].

There are no existing studies that have measured calprotectin concentration in response to iron fortification. Our study showed a significant feeding effect with the addition of CFs, and awe observed the highest calprotectin levels in the Cer + Fr group. Fecal calprotectin concentration has been used to evaluate the degree of inflammation in various systemic and gastrointestinal conditions [36, 37]. Although the current study found that fecal calprotectin concentrations have increased after the introduction of CFs, these elevations remained within the suggested normal values of calprotectin for this age group [38–40]. In a previous study conducted on 74 (39 EBF and 35 formula-fed) term Italian infants, it was reported that the median fecal calprotectin was higher in EBF infants than in formula fed infant (*p* < 0.001) [41]. It was concluded that there was a feeding effect on calprotecin concentration, which might be due to the effect of human milk bioactive molecules, which clearly contribute to the development of the gastrointestinal system [41]. Another study had also indicated that calprotectin is higher in EBF infants than in mixed fed infants [42].

### Fecal microbiota

In agreement with previous studies [18, 43], we found that gut microbiota richness increased after the introduction of CFs. In an observational study, Thompson et al. reported increased richness among infants consuming solid foods, but they did not investigate differences by type of CF. In our study, richness increased after the introduction of meat but no change was observed with iron-fortified cereal. Similar results were reported in a recent RCT of American infants by Krebs et al [18]. In another RCT by Jaeggi et al. [17], iron supplementation was found to adversely affect the gut microbiota of Kenyan infants by increasing the abundance of pathogenic bacteria, including members of the Enterobacteriaceae family. In contrast, we found that Enterobacteriaceae were more abundant in the meat group compared to the cereal groups; however, we did not perform targeted quantitative analyses to identify pathogenic strains. In addition, the difference in the settings between the Kenyan study and ours, such as the poor sanitation and the prevalence of intestinal infections, might have contributed to this variation between our results compared to the Kenyan study. Ours is the first study to evaluate associations between infant gut microbiota and fecal ROS, finding a positive correlation with Coriobacteriaceae. This family from the Actinobacteria phylum is typically elevated in formula versus breast-fed infants and has been linked to host lipid metabolism [44]. Taken together, our results add to existing evidence that iron fortification influences the infant gut microbiota, with potential implications for the developing immune system.

### Limitations

A major strength in the current study is the use of a controlled randomized design, which is considered the gold standard for nutritional intervention. The precise CF given to the caregivers allowed more isolation of the effect of iron from the CFs on the endpoint measures and reduced possible confounding influences from other foods if given. However, some mothers reported feeding their infants other foods or formula (10%). Other limitations of the current study were the variability in the time of the introduction of CFs between the infants (at parents’ discretion) and the short feeding duration. Another limitation was the incomplete collections for urine and fecal samples for all infants, which resulted in variations in the total number of samples analyzed for each outcome. These limitations could have impaired our ability to detect differences between the groups. Although the present study was sufficient in sample size to detect differences in fecal ROS generation, some comparisons of other analytes were underpowered to observe differences between the groups.

## Conclusion

The current RCT highlights clinically important findings to inform infant feeding guideline updates regarding the optimal first CFs. The results of this study do not support the notion that iron fortification causes untoward effects of ROS generation in the intestinal tract of breastfed infants. The current study provides new evidence for the effect of iron-fortified CFs on the developing infant gut microbiota. Further long-term study is required to determine the association between ROS generation in the intestinal tract, inflammatory markers and infant gut microbiota with subsequent health outcomes.

## Additional files

Additional file 1: Table S1. Energy and macronutrient intake before and after introduction of CFs in the three study groups. Description of data: this table presents the total energy, carbohydrates, protein and fat intakes of the infants of each feeding group. The mean intakes of infants were compared between the three groups and within the three groups. (DOCX 71 kb)
Additional file 2: Table S2. Vitamins intake before and after introduction of CFs in the three study groups. Description of data: this table presents the vitamins intake of the infants of each feeding group. The mean intakes of infants were compared between the three groups and within the three groups. (DOCX 89 kb)
Additional file 3: Table S3. Selective minerals intake before and after introduction of CFs in the three study groups. Description of data: this table presents selective minerals intake of the infants of each feeding group. The mean intakes of infants were compared between the three groups and within the three groups. (DOCX 75 kb)

## Abbreviations

Cer: Cereal group
Cer + Fr: Cereal + fruit group
CFs: Complementary foods
EBF: Exclusively breastfed
ID: Iron deficiency
IDA: Iron deficiency anemia
M: Meat group
RCT: Randomized controlled trial
ROS: Reactive oxygen species

## References

[1] Dee DL, Sharma AJ, Cogswell ME, Grummer-Strawn LM, Fein SB, Scanlon KS (2008). Sources of supplemental iron among breastfed infants during the first year of life. Pediatrics.

[2] Canada H (2012). Canadian paediatric society, dietitians of Canada, breastfeeding committee for Canada. Nutrition for healthy term infants: recommendations from birth to six months. Can J Diet Pract Res.

[3] World Health Organization (2001). Global strategy for infant and young child feeding: the optimal duration of exclusive breastfeeding.

[4] Friel JK, Isaak CA, Hanning R, Miller A (2009). Complementary food consumption of Canadian infants. Open Nutr J.

[5] Davidsson L, Kastenmayer P, Szajewska H, Hurrell RF, Barclay D (2000). Iron bioavailability in infants from an infant cereal fortified with ferric pyrophosphate or ferrous fumarate. Am J Clin Nutr.

[6] Grummer-Strawn LM, Scanlon KS, Fein SB (2008). Infant feeding and feeding transitions during the first year of life. Pediatrics.

[7] Lund EK, Wharf SG, Fairweather-Tait SJ, Johnson IT (1999). Oral ferrous sulfate supplements increase the free radical-generating capacity of feces from healthy volunteers. Am J Clin Nutr.

[8] Schümann K, Kroll S, Weiss G, Frank J, Biesalski HK, Daniel H (2005). Monitoring of hematological, inflammatory and oxidative reactions to acute oral iron exposure in human volunteers: preliminary screening for selection of potentially-responsive biomarkers. Toxicology.

[9] Kortman GAM, Raffatellu M, Swinkels DW, Tjalsma H (2014). Nutritional iron turned inside out: intestinal stress from a gut microbial perspective. FEMS Microbiol Rev.

[10] Lotito S, Frei B (2006). Consumption of flavonoid-rich foods and increased plasma antioxidant capacity in humans: cause, consequence, or epiphenomenon?. Free Radic Biol Med.

[11] Orozco MN, Solomons NW, Schumann K, Friel JK, de Montenegro ALM (2010). Antioxidant-rich oral supplements attenuate the effects of oral iron on in situ oxidation susceptibility of human feces. J Nutr.

[12] Halliwell B (1994). Free radicals and antioxidants: a personal view. Nutr Rev.

[13] Halliwell B (1996). Commentary oxidative stress, nutrition and health. Experimental strategies for optimization of nutritional antioxidant intake in humans. Free Radic Res.

[14] Berg R (1996). The indigenous gastrointestinal microflora. Trends Microbiol.

[15] Xavier RJ, Podolsky DK (2007). Unravelling the pathogenesis of inflammatory bowel disease. Nature.

[16] Buhnik-Rosenblau K, Moshe-Belizowski S, Danin-Poleg Y, Meyron-Holtz EG (2012). Genetic modification of iron metabolism in mice affects the gut microbiota. Biometals.

[17] Jaeggi T, Kortman GAM, Moretti D, Chassard C, Holding P, Dostal A (2015). Iron fortification adversely affects the gut microbiome, increases pathogen abundance and induces intestinal inflammation in Kenyan infants. Gut.

[18] Krebs NF, Sherlock LG, Westcott J, Culbertson D, Hambidge KM, Feazel LM (2013). Effects of different complementary feeding regimens on iron status and enteric microbiota in breastfed infants. J Pediatr.

[19] Institute Of Medicine (2001). Dietary reference intakes for vitamin a, vitamin K, arsenic, boron, chromium, copper, iodine, iron, manganese, molybdenum, nickel, silicon, vanadium, and zinc.

[20] Emmett PM, Jones LR (2014). Diet and growth in infancy: relationship to socioeconomic background and to health and development in the Avon longitudinal study of parents and children. Nutr Rev.

[21] Ong KK (2006). Dietary energy intake at the age of 4 months predicts postnatal weight gain and childhood body mass index. Pediatrics.

[22] Kauffman LD, Sokol RJ, Jones RH, Awad JA, Rewers MJ, Norris JM (2003). Urinary F2-isoprostanes in young healthy children at risk for type 1 diabetes mellitus. Free Radic Biol Med.

[23] Davies SS, Zackert W, Luo Y, Cunningham CC, Frisard M, Roberts LJ (2006). Quantification of dinor, dihydro metabolites of F2-isoprostanes in urine by liquid chromatography/tandem mass spectrometry. Anal Biochem.

[24] Owen RW (2000). Generation of reactive oxygen species by the faecal matrix. Gut.

[25] Khafipour E, Li S, Plaizier JC, Krause DO (2009). Rumen microbiome composition determined using two nutritional models of subacute ruminal acidosis. Appl Environ Microbiol.

[26] Caporaso JG, Kuczynski J, Stombaugh J, Bittinger K, Bushman FD, Costello EK (2010). QIIME allows analysis of high-throughput community sequencing data. Nat Methods.

[27] Derakhshani H, Tun HM, Khafipour E (2016). An extended single-index multiplexed 16S rRNA sequencing for microbial community analysis on MiSeq Illumina platforms. J Basic Microbiol.

[28] Masella AP, Bartram AK, Truszkowski JM, Brown DG, Neufeld JD (2012). PANDAseq: paired-end assembler for illumina sequences. BMC Bioinformatics.

[29] DeSantis TZ, Hugenholtz P, Larsen N, Rojas M, Brodie EL, Keller K (2006). Greengenes, a chimera-checked 16S rRNA gene database and workbench compatible with ARB. Appl Environ Microbiol.

[30] Lozupone C, Knight R (2005). UniFrac: a new phylogenetic method for comparing microbial communities. Appl Environ Microbiol.

[31] Schneider BD, Leibold EA (2000). Regulation of mammalian iron homeostasis. Curr Opin Clin Nutr Metab Care.

[32] Finch C (1994). Regulators of iron balance in humans. Blood.

[33] Gibson RS, Bailey KB, Gibbs M, Ferguson EL (2010). A review of phytate, iron, zinc, and calcium concentrations in plant-based complementary foods used in low-income countries and implications for bioavailability. Food Nutr Bull.

[34] Montalbetti N, Simonin A, Kovacs G, Hediger MA (2013). Mammalian iron transporters: families SLC11 and SLC40. Mol Aspects Med.

[35] Kelleher SL, Lönnerdal B (2005). Molecular regulation of milk trace mineral homeostasis. Mol Aspects Med.

[36] Tibble J (2000). A simple method for assessing intestinal inflammation in Crohn’s disease. Gut.

[37] Damms A, Bischoff SC (2008). Validation and clinical significance of a new calprotectin rapid test for the diagnosis of gastrointestinal diseases. Int J Colorectal Dis.

[38] Schoepfer AM, Trummler M, Seeholzer P, Seibold-Schmid B, Seibold F (2008). Discriminating IBD from IBS: comparison of the test performance of fecal markers, blood leukocytes, CRP, and IBD antibodies. Inflamm Bowel Dis.

[39] Olafsdottir E, Aksnes L, Fluge G, Berstad A (2007). Faecal calprotectin levels in infants with infantile colic, healthy infants, children with inflammatory bowel disease, children with recurrent abdominal pain and healthy children. Acta Paediatr.

[40] Fagerberg UL, Lööf L, Merzoug RD, Hansson L-O, Finkel Y (2003). Fecal calprotectin levels in healthy children studied with an improved assay. J Pediatr Gastroenterol Nutr.

[41] Savino F, Castagno E, Calabrese R, Viola S, Oggero R, Miniero R (2010). High faecal calprotectin levels in healthy, exclusively breast-fed infants. Neonatology.

[42] Dorosko SM, MacKenzie T, Connor RI (2008). Fecal calprotectin concentrations are higher in exclusively breastfed infants compared to those who are mixed-fed. Breastfeed Med.

[43] Thompson AL, Monteagudo-Mera A, Cadenas MB, Lampl ML, Azcarate-Peril MA (2015). Milk- and solid-feeding practices and daycare attendance are associated with differences in bacterial diversity, predominant communities, and metabolic and immune function of the infant gut microbiome. Front Cell Infect Microbiol.

[44] Lahti L, Salonen A, Kekkonen RA, Salojärvi J, Jalanka-Tuovinen J, Palva A (2013). Associations between the human intestinal microbiota. Lactobacillus rhamnosus GG and serum lipids indicated by integrated analysis of high-throughput profiling data. PeerJ.

