# Supplementary material for: Assessment of complementary feeding of Canadian infants: effects on microbiome & oxidative stress, a randomized controlled trial
Source: BMC Pediatr. 2017 Feb 14;17:54. doi: 10.1186/s12887-017-0805-0 (PMC5310014; doi:10.1186/s12887-017-0805-0)
Supplement: Additional file 1: Table S1. — Energy and macronutrient intake before and after introduction of CFs in the three study groups. Description of data: this table presents the total energy, carbohydrates, protein and fat intakes of the infants of each feeding group. The mean intakes of infants were compared between the three groups and within the three groups. (DOCX 71 kb) [file 12887_2017_805_MOESM1_ESM.docx]

Table S1. Energy and macronutrient intake before and after introduction of CFs in the three study groups

| **Feeding group**  **Nutrients** (mean±SE) | Before (BM only)  **Cer**   **Cer+Fr**  **(n=22) (n=28)** | | | **M**  **(n=32)** | **All groups**  **(n=82)** | After (BM + Solids)  **Cer**  **Cer+Fr**  **(n=22) (n=28)** | | | **M**  **(n=32)** | | **All groups**  **(n=82)** |
| --- | --- | --- | --- | --- | --- | --- | --- | --- | --- | --- | --- |
| Energy (Kcal) | 647±29.0 | 677±29.7 | 696±30.4 | | 673±17.2 | 776±52.5^a^ | 895±45.8^a^ | 694±36.4 | | 788±27.2 | |
| Energy (KJ) | 2667±144 | 2825±129 | 2896±681 | | 2799±72 | 3248±201^a^ | 3747±180^a^ | 2889±176 | | 3292±114 | |
| Carbohydrate (g) | 63.7±2.8 | 66.6±2.9 | 68.6±2.9 | | 66.5±1.7 | 93.3±6.9^a^ | 110±7.7^a^ | 63.4±3.5^a,b^ | | 88.2±4.2 | |
| Protein (g) | 9.5±0.42 | 9.9±0.43 | 10.2±0.44 | | 9.9±0.25 | 15.5±1.2^a^ | 16.2±1.1^a^ | 14.4±0.81^a^ | | 15.3±0.60 | |
| Fat (g) | 40.5±1.8 | 42.3±1.8 | 43.6±1.9 | | 42.3±1.0 | 39.2±2.7 | 44.0±1.7 | 43.8±2.3 | | 42.6±1.3 | |

N.B: ^a^ values superscript letters are significantly different intake over time (*p* < 0.05), ^b^ values superscript letters are significantly different between the groups
